# Rating Communication in GP Consultations: The Association Between Ratings Made by Patients and Trained Clinical Raters

**DOI:** 10.1177/1077558716671217

**Published:** 2016-10-03

**Authors:** Jenni Burt, Gary Abel, Natasha Elmore, Jenny Newbould, Antoinette Davey, Nadia Llanwarne, Inocencio Maramba, Charlotte Paddison, John Benson, Jonathan Silverman, Marc N. Elliott, John Campbell, Martin Roland

**Affiliations:** 1 University of Cambridge School of Clinical Medicine, Cambridge, UK; 2 University of Exeter Medical School, Exeter, Devon, UK; 3 Anglia Ruskin University, Cambridge, UK; 4 RAND Corporation, Santa Monica, CA, USA

**Keywords:** patient experience, physician–patient communication, health care surveys, health care quality measurement, health care quality

## Abstract

Patient evaluations of physician communication are widely used, but we know little about how these relate to professionally agreed norms of communication quality. We report an investigation into the association between patient assessments of communication quality and an observer-rated measure of communication competence. Consent was obtained to video record consultations with Family Practitioners in England, following which patients rated the physician’s communication skills. A sample of consultation videos was subsequently evaluated by trained clinical raters using an instrument derived from the Calgary-Cambridge guide to the medical interview. Consultations scored highly for communication by clinical raters were also scored highly by patients. However, when clinical raters judged communication to be of lower quality, patient scores ranged from “poor” to “very good.” Some patients may be inhibited from rating poor communication negatively. Patient evaluations can be useful for measuring relative performance of physicians’ communication skills, but absolute scores should be interpreted with caution.

## Introduction

Good physician–patient communication is central to good patient experience, and a major driver of overall patient assessments of primary care in the United States and the United Kingdom (Paddison et al., 2013; Quigley et al., 2014). Communication skills are consequently core strands of medical training, postgraduate assessment, and ongoing professional development (Accreditation Council for Graduate Medical Education, 2015; General Medical Council, 2015). While communication is important in its own right, it may be associated with other dimensions of quality of care such as clinical effectiveness and patient safety, and evidence suggests that good communication skills tend to be found alongside good clinical skills (Doyle, Lennox, & Bell, 2013; Llanwarne et al., 2013; Price, Elliott, Cleary, Zaslavsky, & Hays, 2015). The quality of physician–patient communication has been associated with patient adherence to treatment (Zolnierek & Dimatteo, 2009), uptake of cancer screening (Carcaise-Edinboro & Bradley, 2008), improved blood pressure control in hypertensive patients (Orth, Stiles, Scherwitz, Hennrikus, & Vallbona, 1987), and reductions in the risk of serious medical error (Kuzel et al., 2004). However, concerns about the quality of physician–patient communication remain, and a significant proportion of malpractice claims are driven by poor communication (Tamblyn et al., 2007; Vincent et al., 2006).

The quality of physician–patient communication is assessed using approaches including observer rating and patient surveys (Duffy et al., 2004). Observer rating of consultation skills relies on either the use of simulated patients, such as in Objective Structured Clinical Exams (Turner & Dankoski, 2008), or in the observation or videotaping of actual consultations (encounters; Zill et al., 2014). Due to its complexity, observer rating is usually confined to the assessment of medical students and postgraduate examinations, or used for research purposes. Patient surveys, by contrast, are widely used to assess the standard of physician–patient communication, and national survey programs include the English GP Patient Survey and the U.S. CAHPS (Consumer Assessment of Healthcare Providers and Systems; Agency for Healthcare Research and Quality, 2015; Ipsos MORI, 2015). Findings from such surveys inform a variety of official metrics of care quality. In England, the Care Quality Commission (the regulator of health and social care) uses data from the GP Patient Survey as part of its monitoring of key performance indicators in its practice inspection regime (Care Quality Commission, 2015). Additionally, GP Patient Survey data are made publicly available both via a dedicated website (https://gp-patient.co.uk) and in the form of performance scores attached to practice profiles on a public listing of NHS services (http://www.nhs.uk). In the United States, CAHPS scores influence payments to hospitals and Medicare plans (Centers for Medicare & Medicaid Services, 2012; Medicare, 2016).

### New Contributions

Understanding the meaning of such patient assessments of care within the context of accepted professional standards is crucial to the expectation that patient feedback can and should act as a catalyst to change. Previous research has explored the relationship between patient and examiner ratings of trainee general practitioner (GP) communication skills, and has found either no evidence of an association (in an underpowered study, with a sample size of 19 [McKinstry, Walker, Blaney, Heaney, & Begg, 2004]) or weak-to-moderate association (Greco, Spike, Powell, & Brownlea, 2002). More recently, a study of observer-rated verbal and nonverbal elements of a consultation found aspects of these predicted patient satisfaction with communication and the doctor–patient relationship (Little et al., 2015). However, no study has yet explored the association between patient assessments of communication skills on items used in national survey programs (and consequent quality metrics) and observer assessment of the performance of practicing physicians.

This study investigated the association between patient assessments of the quality of communication in their consultations with their Family Physician, using items derived from the English GP Patient Survey (Ipsos MORI, 2015), and a recently developed observer-rated measure of communication competence, the Global Consultation Rating Scale (GCRS), derived from the Calgary-Cambridge guide to the medical interview (Burt et al., 2014; Kurtz & Silverman, 1996; Kurtz, Silverman, Benson, & Draper, 2003).

## Conceptual Model

**Figure 1.**
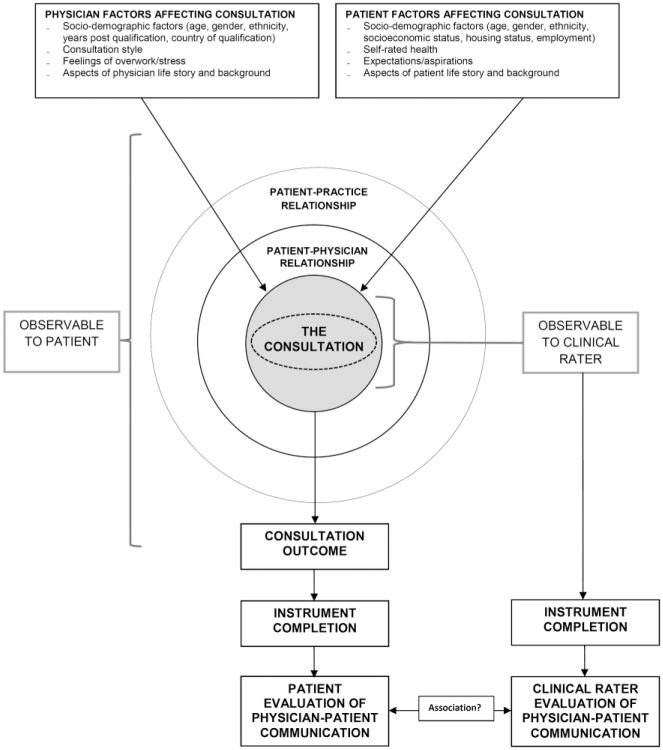
Conceptual model: mechanisms of patient and clinical rater assessment of physician-patient communication in a consultation.

In this study, we hypothesized that patient evaluations of physician communication, as expressed on patient experience instruments, may be influenced by a number of factors both internal and external to the consultation (see Figure 1). Some of these will be, and some will not be, visible to an outside observer. For example, both the patient and the physician they are consulting with bring various characteristics and experiences to each consultation which will determine the interaction. These come together as the overall “consultation experience,” which is nested within both the previous physician–patient relationship (if there is one), and the relationship the patient has with the wider practice. These, together with the outcome of the consultation, will determine the patient’s evaluation of physician communication. Thus, while an instrument may ask patients to rate their experience of being listened to or involved in decision making in a particular consultation, their choice of answer could be influenced by a host of other factors as well as these particular dimensions of care. For example, the same physician communication may produce different patient experiences in patients with different health literacy. An external observer, by contrast, is unlikely to know anything about the patient’s past history with the practice or their characteristics and experiences, other than that which is directly observable through the consultation. This limits the observer to rating to what can be seen and judged from the consultation itself. This is not to say that observers are free from influence, far from it, and variability in raters’ assessments of the same clinical consultation is a well-known phenomenon (Burt et al., 2014). Through such mechanisms, we anticipated that it was possible that patient and clinical rater assessments may not always align, as patients and raters may be assessing related but distinct constructs. While raters assess the extent to which physicians’ communication adheres to best practices, patients report on the effects of that communication on their health care experiences.

## Method

The study was conducted in General Practices in England in two broad geographic areas (Devon, Cornwall, Bristol, Dorset, and Somerset; and Cambridgeshire, Bedford, Luton, and North London). Practices were eligible if they (a) had more than one Family Physician (hereafter just “physician”) working a minimum of 2 days per week in direct clinical contact with patients and (b) had low scores on physician–patient communication items used in the national GP Patient Survey (defined as practices below the 25th percentile for mean communication score in the 2009/2010 survey, adjusted for patient case mix [Paddison et al., 2012]). Low-scoring practices were chosen to obtain more consultations with low patient ratings for communication than is typical: Nationally, 94% of patients score all questions addressing GP communication within consultations as good or very good (the two most positive options) in the GP Patient Survey.

### Recruitment

In order to obtain the 28 required video recorded consultations that patients judged to have less than good communication (see below), many more consultations had to be video recorded than would be rated. The 28 required “good” consultations were more easily obtained, as they were more common. The research team approached adult patients on their arrival in participating practices and sought written informed consent to video record their consultation. Data collection continued until each required number was reached.

### Patient Ratings

Immediately following the consultation the patient was asked to complete a short questionnaire. The questionnaire included a set of seven items taken from the national GP Patient Survey to assess physician–patient communication (Box 1), and basic sociodemographic questions. The seven patient communication items were previously derived to represent a single underlying construct; this has been confirmed using factor analysis (Campbell et al., 2009). As previously, we calculated a physician–patient communication score by linearly rescaling responses between 0 and 100 and taking the mean of all responses where four or more informative answers were given (Lyratzopoulos et al., 2012; Roberts et al., 2014).

**Box 1.** GP-Patient Communication Items Used in the Patient Experience Survey.

Thinking about the consultation which took place *today*						
**How good was the doctor at each of the following?**						
**Please put an ✘ in one box for *each* row.**						
	**Very good**	**Good**	**Neither good nor poor**	**Poor**	**Very poor**	Doesn’t apply*
Giving you enough time . . .	□	□	□	□	□	□
Asking about your symptoms . . .	□	□	□	□	□	□
Listening to you . . .	□	□	□	□	□	□
Explaining tests and treatments . . .	□	□	□	□	□	□
Involving you in decisions about your care	□	□	□	□	□	□
Treating you with care and concern . . .	□	□	□	□	□	□
Taking your problems seriously . . .	□	□	□	□	□	□

* Considered to be uninformative for the purposes of our analysis.

### Ratings by Trained Clinical Raters

We sampled 56 consultations for rating by experienced trained clinical raters. Raters scored each of the selected consultations using the GCRS (Burt et al., 2014). The GCRS is designed to assess the effectiveness of communication across an entire consultation, and is based on the widely used Calgary-Cambridge guide to the medical interview (Kurtz et al., 2003; Kurtz & Silverman, 1996). Raters score each consultation in 12 domains (including gathering information, building the relationship, providing structure, and achieving a shared understanding), resulting in a final score of between 0 and 10 (see supplementary material for full instrument [all supplementary materials are available online at http://mcr.sagepub.com/content/by/supplemental-data]). Raters were physicians experienced in the teaching of communication skills; all attended a 2-hour training session on GCRS delivered by one of the original authors of the Calgary-Cambridge guide (JS). We used four raters for each consultation to increase reliability. Each rater scored consultations in a different random order to minimize the consequences of any order effects, and the same raters were used for all consultations. A simple mean of the four raters was calculated for each consultation.

From the rating of 56 consultations, we expected 80% power (.05 significance level) to detect a correlation coefficient of .37. To best measure this correlation our a priori sampling strategy included consultations with a wide range of scores: 28 (half) from those where all patient responses to the seven communication items were either *good* or *very good*, and 28 (half) where at least one rating was less than *good*. For the 28 “less-than-good” consultations, we selected the 28 consultations with the lowest patient communication scores. The 28 “good” consultations were selected at random. We further barred the inclusion of more than two consultations involving the same physician.

### Statistical Analyses

First, we assessed the rater-adjusted consultation-level reliability of the GCRS scores by fitting a mixed-effects linear regression model to the 224 individual ratings (four ratings of 56 consultations). Following the standard approach for adjusted unit-level reliability (e.g., Elliott et al., 2010), we included a random consultation effect for between-consultation variance ($\sigma_{b}^{2}$) and rater fixed effects, with the residual capturing within-consultation, between-rater, variance ($\sigma_{w}^{2}$) in ratings. The reliability ($\lambda_{GCRS}$) of the mean GCRS rating is as follows:

$$\lambda_{GCRS}=\frac{\sigma_{b}^{2}}{\sigma_{b}^{2}+\frac{\sigma_{w}^{2}}{4}}$$

We explored the association between individual patient ratings and the mean ratings obtained by four trained raters using a simple correlation coefficient and scatter plot. Because adjusting for patient sociodemographic characteristics did not meaningfully reduce standard errors, unadjusted results are shown. Bootstrapping with 1,000 replicates, clustered within physicians, accounted for some physicians being included twice and for possible deviations from normality. Further illustration was provided by dichotomizing patient ratings into below 75 (requiring at least one less-than-good response) versus higher and cross-tabulating this with tertiles of GCRS ratings. The resulting 2 × 3 association was tested using logistic regression with a sandwich estimator to account for clustering by physician (Rogers, 1993).

The data analyzed relate to patient ratings of a particular consultation, rather than many patients’ ratings of a particular physician. Even when the association between patient scores and rater scores is weak, it may be that by aggregating scores from many patients, reliable physician scores may be obtained. We illustrate this concept by simulating scores for 100 hypothetical physicians with a range of communication skills as measured by GCRS. The patient ratings for a given GCRS score are drawn from a distribution informed by the findings of the observational work. For each physician, mean patient scores are calculated for 1, 10, 30, and 100 patients. In this illustration physicians are assumed to score consistently on GCRS for all consultations.

All analysis was carried out using Stata V13.1 (StataCorp, 2015, Stata Statistical Software: Release 13, College Station, TX).

## Results

**Figure 2.**
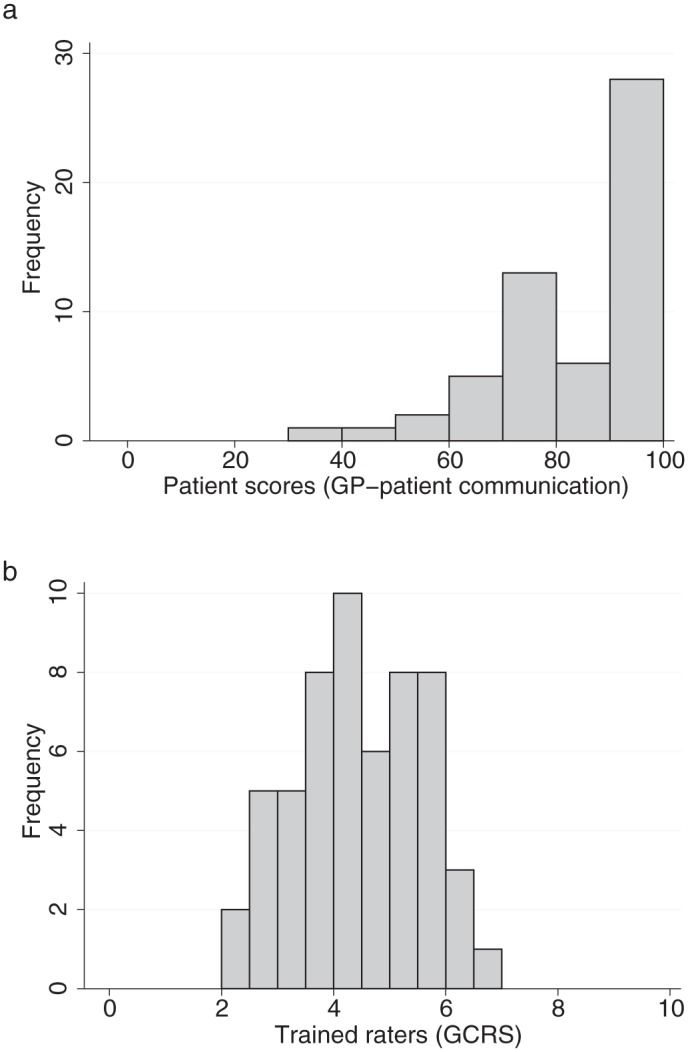
Distribution of patient scores based on GP patient survey items and ratings by trained raters on the GCRS. *Note*. GCRS = Global Consultation Rating Scale.

Consultations with 45 participating physicians from 13 general practices were video recorded. Of 741 eligible patients, 529 (71.4%) consented to participate and completed a questionnaire (see supplementary material for a recruitment flowchart). Reasons patients gave for declining participation commonly related to the nature of their clinical problem. The videos selected for rating using GCRS came from all 13 general practices and included 37 physicians. Table 1 shows the self-reported demographics of patients who completed a questionnaire, along with those whose consultation was selected for rating by trained raters. Men, 18- to 24-year-olds, and Asian patients were somewhat more likely to have been selected to have their consultations rated. The distribution of patient scores and GCRS ratings is shown in Figure 2. Patient scores were highly skewed: The most common score (found for 21/56 consultations) was 100 out of a possible 100 (i.e., *very good* for all reported communication items). The median score was 91 (interquartile range 71-100) and the lowest reported score 31. In contrast, the GCRS ratings are reasonably symmetrical: The median GCRS score was 4.3 of 10 (interquartile range 3.6-5.5) and scores ranged from 2.2 to 6.8. The estimated variance components of the GCRS ratings were 1.01 between consultations and 1.18 within consultations (between rater). Reliability for the mean of four ratings was 0.77.

**Table 1.** Self-Reported Demographics for Patients Who Completed a Questionnaire and Those Selected for Consultations to be Rated by Trained Raters.

	Completed questionnaire, *n* (%)	Rated consultations, *n* (%)
Sex		
Male	212 (40.15)	26 (46.43)
Female	316 (59.85)	30 (53.57)
Age, years		
18-24	39 (7.41)	10 (18.18)
25-34	78 (14.83)	7 (12.73)
35-44	64 (12.17)	7 (12.73)
45-54	82 (15.59)	4 (7.27)
55-64	85 (16.16)	8 (14.55)
65-74	103 (19.58)	7 (12.73)
75-84	60 (11.41)	8 (14.55)
85+	15 (2.85)	4 (7.27)
Self-rated health		
Excellent	50 (9.51)	3 (5.36)
Very good	173 (32.89)	14 (25)
Good	182 (34.60)	23 (41.07)
Fair	83 (15.78)	13 (23.21)
Poor	38 (7.22)	3 (5.36)
Ethnicity		
White	474 (90.98)	44 (81.48)
Mixed	5 (0.96)	1 (1.85)
Asian or Asian British	15 (2.88)	6 (11.11)
Black or Black British	22 (4.22)	1 (1.85)
Chinese	4 (0.77)	1 (1.85)
Other	1 (0.19)	1 (1.85)

**Figure 3.**
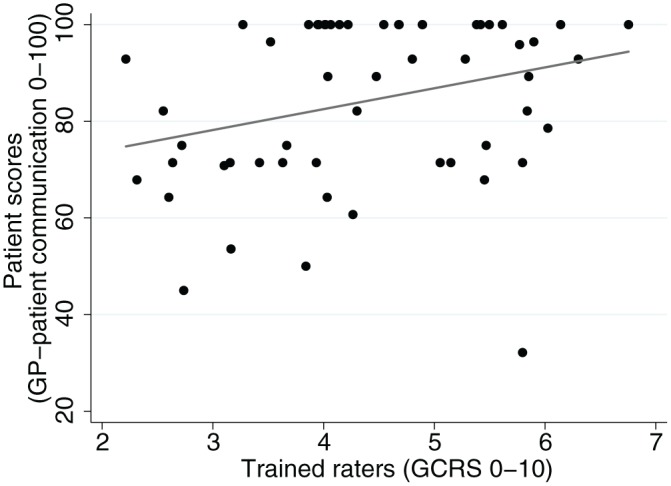
Scatterplot comparing patient scores based on GP patient survey items and ratings by trained raters on the GCRS. *Note*. GCRS = Global Consultation Rating Scale.

Figure 3 shows patient scores plotted against average GCRS ratings for each consultation. There is weak evidence (*p* = .054) of an association between patient scores and GCRS ratings, with an unadjusted correlation coefficient of .29 (reliability-adjusted *r* = .33; Muchinsky, 1996). When trained raters assessed communication within a consultation to be of a high standard (highest tertile), patients tended to do the same (with the exception of a single outlying low patient score). However, when trained raters judged communication within a consultation to be poor (lowest tertile), patients reported communication ranging from poor to very good. This is illustrated in Table 2, which shows that in the consultations in the lowest third of rater scores 58% of patient scores were under 75 (out of 100) compared with 17% in the highest third.

**Table 2.** Comparison of Lower (<75) and Higher (≥75) Patient Scores for Consultations in Each Third of the Distribution of Rater Scores.

	Patient score (GP–patient communication, 0-100 scale)		
	<75, *n* (%)	≥75, *n* (%)	Total, *n* (%)
Trained raters (GCRS 0-10 scale)			
Lowest third	11 (57.9)	8 (42.1)	19 (100)
Middle third	4 (21.1)	15 (79.0)	19 (100)
Highest third	3 (16.7)	15 (83.3)	18 (100)
Total	18 (32.1)	38 (67.9)	56 (100)

*Note*. GCRS = Global Consultation Rating Scale. Test of association from logistic regression accounting for clustering by physician, *p* = .049.

**Figure 4.**
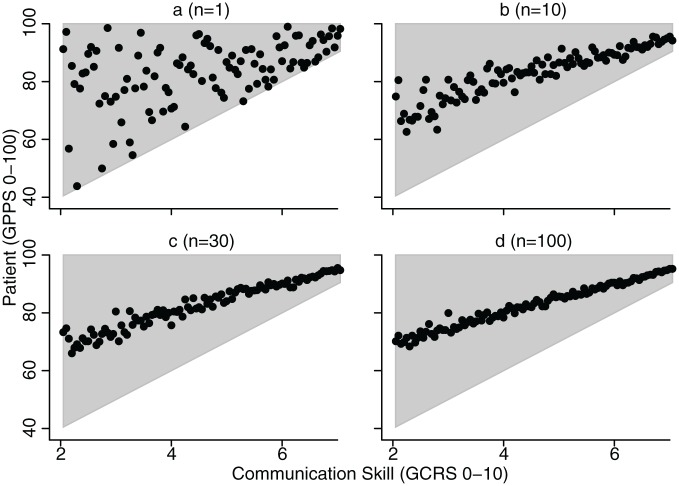
Results of simulation illustrating the effect of estimating physician communication scores based on different numbers of patient ratings. In the simulation the range of individual patient sores we allowed to be taken for any one value of communication skill are shown by the grey areas. The black points show the scores for individual simulated physicians when averaging over 1 (a), 10 (b), 30 (c) and 100 (d) patient scores for each physician. *Note*. GCRS = Global Consultation Rating Scale.

Figure 4 shows the results of the simulation study, which is based on a hypothetical set of consultations with a range of trained rater scores (GCRS). For each GCRS score, we defined a range of possible simulated patient scores, shown by the shaded grey area in Figure 4. The lower limit of these simulated patient scores increased as GCRS score increased. However, the upper limit of simulated patient scores was set at 100 for all possible GCRS scores in the simulation. For any given GCRS score, we allowed patient scores to take any value in this range, with equal probability. The simulation is designed for illustrative purposes only and is not intended to directly reflect our current findings. Panel A, designed to be reminiscent of Figure 3, shows what would be observed with just a single patient score per physician, that is, a weak correlation between patient rating and communication skill. The remaining panels illustrate the effect of combining scores (taking the mean) from multiple consultations, rather than using a single rating. As the number of patient ratings taken increases, the correlation between trained rater scores and patient scores gets stronger. When the number of consultations are 30, this correlation becomes very strong (ρ = .97), becoming stronger still when *n* = 100.

## Discussion

This study aimed to compare patient assessments of physician communication with measures of adherence to professionally agreed standards and norms of physician communication. We found a weak positive correlation between patients’ and trained clinical raters’ assessments of the quality of communication in physician–patient consultations. This suggests that there is an association between patient ratings of communication and professionally defined standards of care. Importantly, when trained clinical raters identified communication as good, patients tended to agree with this. However, when trained clinical raters identified communication as poor, patients ranged in their assessments of communication from poor to very good.

National survey programs commonly feedback patients’ evaluations to physicians, assuming that mechanisms such as reputational concern will drive performance improvements (Contandriopoulos, Champagne, & Denis, 2014; Fung, Lim, Mattke, Damberg, & Shekelle, 2008). However, recent evidence suggests that physicians struggle to make sense of patient experience feedback and may dismiss findings rather than initiate change (Asprey et al., 2013; Boiko et al., 2014). The persistence of concerns about the reliability and validity of the questionnaires used are key factors contributing toward disregard for patient survey results (Boiko et al., 2014). These concerns are complex, and driven in part by limited information regarding how much patient ratings: (a) confined to perceive physician communication quality alone, without being influenced by other considerations or aspects of care or (b) match accepted professional standards of communication. Our study aimed to address the latter question, and our findings suggest that, while trained raters’ and patient’s tend to agree what good communication looks like in a consultation, clinical raters are more likely than patients to judge communication as poor.

We outline two possible mechanisms driving this divergence: In both cases, we are benchmarking the patient ratings against those of the raters (an issue we discuss further below). The first mechanism arises from the well-known phenomenon of skewed patients’ ratings, or positive response tendency, with a large proportion of patients rating communication as “very good” (Campbell et al., 2009; Elliott, Haviland, Kanouse, Hambarsoomian, & Hays, 2009; Rodriguez & Crane, 2011). By contrast, GCRS ratings tend to cluster around the middle scores available to raters. It is therefore possible that the poor measurement of above-average experiences inherent in the patient question items may artificially limit the responses patients would like to give (thereby applying a ceiling effect), preventing them from being able to distinguish the very best consultations from those they judge to be simply good. This mechanism requires that patients differ from raters in their views of what good or poor communication in a consultation looks like. As a result, the more positive patient opinion is “held back” by only being able to endorse questionnaire options ranging from very poor to very good (and not, e.g., “excellent”), despite extensive instrument development (Ipsos MORI, 2015).

However, the second (and we argue more plausible) mechanism is that there are wider factors at play which inhibit some patients from assigning poor scores to consultations that they do perceive as involving poor communication. It is important to note that any such inhibition would have to apply unevenly between patients to explain the range of patient scores seen for consultations rated as poor by the trained raters: While some patients are easily able to choose “poor” as an option, others feel less able to do so. This is distinct from the ceiling effect described above in so far as consultations are not being underrated due to the maximum available rating, but that ratings are often higher than the consultation might merit.

While we are unable to determine the relative contribution of either mechanism from the methodology of this current study, there is existing evidence that patients may be inhibited in their judgments of care. This hypothesis is consistent with evidence that tendencies to avoid negative and extreme responses vary across patients (Elliott et al., 2009; Mayer, Elliott, Haas, Hays, & Weinick, 2016). In addition, qualitative research has identified a number of psychological and social factors that suggest patients struggle to criticize physicians’ performance in surveys. For example, an investigation into how patients evaluated community mental health services found that they frequently avoided giving negative scores on experience questionnaires: Instead, allowances for poor care were constructed by referencing their perceptions of the duties and culpabilities of health care providers (Williams, Coyle, & Healy, 1998). Similarly, patients undergoing elective orthopedic surgery reinterpreted their experiences in a positive light as a result of feelings of dependency on their health care providers, and a perceived need to maintain constructive relationships with physicians (Edwards, Staniszweska, & Crichton, 2004). A tendency to excuse rather than report poor care has also been identified in breast cancer patients (Davoll et al., 2013). In light of this, we are currently undertaking further qualitative research with our sample to determine whether such factors may be found within the assessment of patient experience in primary care.

### Limitations

Our sampling strategy was informed by the need to locate consultations patients identified as less than good; the proportion of such consultations is small, so to increase study efficiency, we deliberately approached some practices who had received lower scores for communication in the national GP Patient Survey. Not all physicians in every practice took part, and it is possible that the physicians who participated were more confident in their ability to communicate with patients. If those physicians who had poorer communication skills did not participate, this may have reduced the variability of the communication quality in our sample, in turn reducing study power and, potentially, the strength of the observed correlation. Power was also limited by the number of consultations rated and, while the study was not powered to detect weak correlations, it did have sufficient power to detect moderate ones.

Our patient consent rate was 71.4% of eligible patients. The research team missed only a small number of patients (2.0% [15/741] of those eligible), so exclusions predominantly reflect those who did not consent to participate. Recorded consultations concerning some medical conditions may be underrepresented as participants may have been more likely to decline being video recorded: While we were not able to elicit detailed reasons from patients who declined to participate, our observations suggest that in some cases this was due to sensitive presenting complaints. However, participants’ age, gender, self-rated health, and ethnicity were broadly representative of the population attending general practice.

We assessed communication using two well-validated instruments: the GP Patient Survey items for patients and the GCRS for trained raters (Burt et al., 2014; Ipsos MORI, 2015). The GCRS was derived from the Calgary-Cambridge guide, which is used widely for communication skills training, and represents agreed professional norms of high-quality communication (Gillard, Benson, & Silverman, 2009; Kurtz et al., 2003; Kurtz & Silverman, 1996). Recently, the question has arisen as to how and whether trained raters take account of contextual factors in assessing the communication skills of physicians, for example, by allowing variations from “accepted practice” when scoring performance in particular situations (Essers et al., 2013; Essers et al., 2014). However, the GCRS has been explicitly designed to focus only on the consultation process, and contains no task-based items which may be context-specific. Additionally, it enables raters to choose “not applicable” where necessary: In fact, this was rarely endorsed by raters in this study.

As mentioned above, in drawing conclusions about the meaning of patients’ ratings of communication quality, we compare them with assessments by trained clinical rater. This is not to suggest raters are more valued or competent assessors of communication than patients, but simply to use them as representative of professionally agreed norms of behavior against which to judge patient evaluations of communication. In doing so, we are able to provide evidence that to some extent patient assessments tap in to the same underlying construct of communication drawn on by trained raters, but also that patients are less likely to judge consultations as poor.

### Conclusions

Patient experience surveys are widely used to assess the standard of care provision. While physicians rated poorly by patients are generally rated poorly by trained raters, our findings suggest that patients may be inhibited in criticizing doctors’ performances. Mean patient survey scores are likely to overestimate adherence to best physician communication practices, and treating apparently high patient experience scores as indicating absolutely high physician or practice performance is inadvisable. However, the use of relative rankings to identify physicians who are better or poorer at communicating with patients may be an acceptable approach to benchmarking performance, as long as statistically reliable figures are obtained. Previous research has demonstrated that the GP Patient Survey communication questions can differentiate between the performance of physicians and practices, as long as an adequate sample size is used to achieve acceptable statistical reliability (Lyratzopoulos et al., 2011; Roberts et al., 2014). This was confirmed by our simulation: With sufficient patient scores, a strong correlation between patient rating and rater evaluations will be observed. In the use of patient experience scores as quality indicators, our findings suggest that it is therefore possible to (a) trust aggregated patients scores that meet traditional standards of reliability as valid measures of comparative performance with respect to communication and (b) trust relatively low mean patient ratings as indicating poor performance. However, crucially, we cannot necessarily assume that an apparently high mean patient rating means all is well. Thus, lower patient experience scores should spur improvement efforts and higher scores should not breed complacency.

## Supplemental Material

Supplementary_material_Med_Care_Res_Rev – Supplemental material for Rating Communication in GP Consultations: The Association Between Ratings Made by Patients and Trained Clinical Raters Click here for additional data file. Supplemental material, Supplementary_material_Med_Care_Res_Rev for Rating Communication in GP Consultations: The Association Between Ratings Made by Patients and Trained Clinical Raters by Jenni Burt, Gary Abel, Natasha Elmore, Jenny Newbould, Antoinette Davey, Nadia Llanwarne, Inocencio Maramba, Charlotte Paddison, John Benson, Jonathan Silverman, Marc N. Elliott, John Campbell, and Martin Roland in Medical Care Research and Review
