# Supplementary material for: Rating Communication in GP Consultations: The Association Between Ratings Made by Patients and Trained Clinical Raters
Source: Med Care Res Rev. 2016 Oct 3;75(2):201–18. doi: 10.1177/1077558716671217 (PMC5858640; doi:10.1177/1077558716671217)
Supplement: Supplementary_material_Med_Care_Res_Rev – Supplemental material for Rating Communication in GP Consultations: The Association Between Ratings Made by Patients and Trained Clinical Raters [file Supplementary_material_Med_Care_Res_Rev.docx]

**SUPPLEMENTARY/APPENDIX MATERIAL**

| **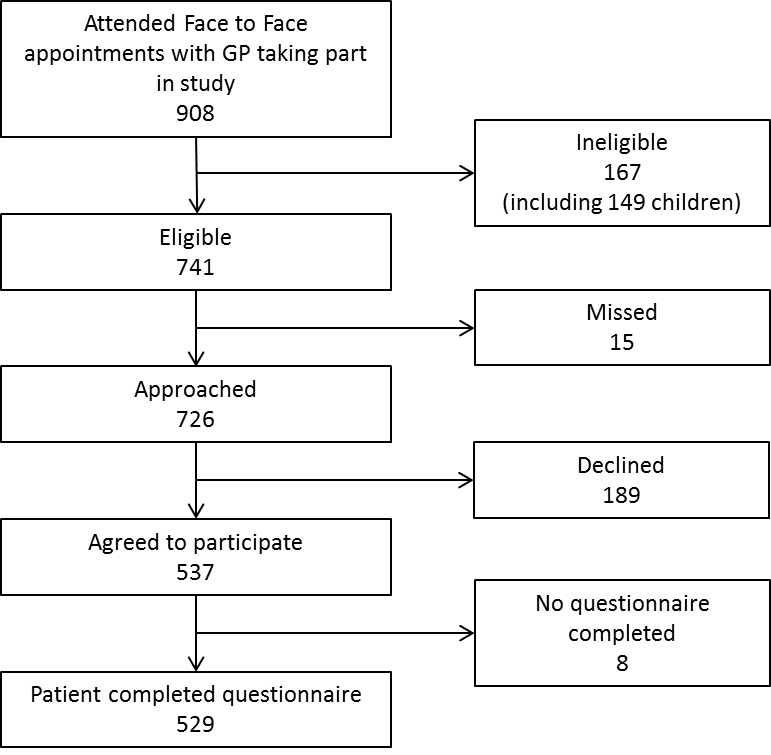** |
| --- |
| **Flow chart illustrating the recruitment and participation of patients.** |
